# A Differential Wiring Analysis of Expression Data Correctly Identifies the Gene Containing the Causal Mutation

**DOI:** 10.1371/journal.pcbi.1000382

**Published:** 2009-05-01

**Authors:** Nicholas J. Hudson, Antonio Reverter, Brian P. Dalrymple

**Affiliations:** Food Futures Flagship and Livestock Industries, Commonwealth Scientific and Industrial Research Organisation, Queensland Bioscience Precinct, St. Lucia Brisbane, Queensland, Australia; The Hebrew University, Israel

## Abstract

Transcription factor (TF) regulation is often post-translational. TF modifications such as reversible phosphorylation and missense mutations, which can act independent of TF expression level, are overlooked by differential expression analysis. Using bovine Piedmontese myostatin mutants as proof-of-concept, we propose a new algorithm that correctly identifies the gene containing the causal mutation from microarray data alone. The myostatin mutation releases the brakes on Piedmontese muscle growth by translating a dysfunctional protein. Compared to a less muscular non-mutant breed we find that myostatin is not differentially expressed at any of ten developmental time points. Despite this challenge, the algorithm identifies the myostatin ‘smoking gun’ through a coordinated, simultaneous, weighted integration of three sources of microarray information: transcript abundance, differential expression, and differential wiring. By asking the novel question “which regulator is cumulatively most differentially wired to the abundant most differentially expressed genes?” it yields the correct answer, “myostatin”. Our new approach identifies causal regulatory changes by globally contrasting co-expression network dynamics. The entirely data-driven ‘weighting’ procedure emphasises regulatory movement relative to the phenotypically relevant part of the network. In contrast to other published methods that compare co-expression networks, significance testing is not used to eliminate connections.

## Author Summary

Evolution, development, and cancer are governed by regulatory circuits where the central nodes are transcription factors. Consequently, there is great interest in methods that can identify the causal mutation/perturbation responsible for any circuit rewiring. The most widely available high-throughput technology, the microarray, assays the transcriptome. However, many regulatory perturbations are post-transcriptional. This means that they are overlooked by traditional differential gene expression analysis. We hypothesised that by viewing biological systems as networks one could identify causal mutations and perturbations by examining those regulators whose position in the network changes the most. Using muscular myostatin mutant cattle as a proof-of-concept, we propose an analysis that succeeds based solely on microarray expression data from just 27 animals. Our analysis differs from competing network approaches in that we do not use significance testing to eliminate connections. All connections are contrasted, no matter how weak. Further, the identity of target genes is maintained throughout the analysis. Finally, the analysis is ‘weighted’ such that movement relative to the phenotypically most relevant part of the network is emphasised. By identifying the question to which myostatin is the answer, we present a comparison of network connectivity that is potentially generalisable.

## Introduction

Evolution, normal development, immune responses and aberrant processes such as diseases and cancer all involve at least some rewiring of regulatory circuits [1]–[3]. Indeed it is the subtle (and sometimes not so subtle) differences in circuit wiring that makes each individual unique. The key nodes in regulatory circuits are frequently transcription factors (TF) [4]. Thus, there is a great deal of interest in developing methods for decoding TF changes. Regulator-target interactions can be assessed by ChIP-on-chip but this requires large amounts of homogenous starting material and TF-specific reagents. Furthermore, the recruitment of a TF to a promoter does not necessarily correlate with transcriptional status, so biological interpretation can be complex [5]. Likely sites of key regulatory mutations can be revealed by Whole Genome Scans (WGS) but this approach requires large numbers of individuals and very dense SNP panels. Even so, the exact causal gene may remain ambiguous if there are several genes near the marker. In any case, little insight is gained into the underlying regulatory mechanisms. In order to gain further insights into the regulatory apparatus, computational approaches are continuously being proposed. To date, they all operate by integrating information from multiple levels of biological organisation particularly eQTL, protein-protein interaction and TF binding site data [6]–[9].

Identifying regulatory change solely through contrasts in gene expression data has been elusive because TF tend to be stably expressed at baseline levels [10] close to the sensitivity of standard high-throughput expression profiling platforms. Further, TF activation is often regulated post-translationally and thereby can act somewhat independently of expression level. Biologically important common TF activation processes (localisation to the nucleus, phosphorylation, ligand binding, formation of transcriptionally ‘open’ euchromatin, and presence of cofactors, all in addition to mutations in the protein coding region of the regulator) are poorly detected by conventional differential expression (DE) analysis.

We hypothesised that a system-wide network approach might have utility, on the grounds that while a differentially-regulated TF might not be DE between two systems, its new position in the network of the perturbed system might allow detection of the ‘smoking gun.’ To allow reliable evaluation of such a hypothesis a well-defined experimental model system is required. Piedmontese cattle are double-muscled because they possess a genomic DNA mutation in the myostatin (GDF8) mRNA transcript [11]. The resulting dysfunctional myostatin protein is a transcriptional regulator that releases the brakes on muscle growth reflecting the importance of TGF-β signalling pathways in the determination of final muscle mass and fibre composition [12],[13]. A preliminary analysis of the expression of myostatin in Piedmontese×Hereford versus Wagyu×Hereford animals found that DE of myostatin was not detectable using cDNA-based expression microarrays [14],[15].

Thus we have a system in which we know the identity of the gene containing the causal mutation, myostatin (MSTN), but we cannot identify it by DE of the mRNA in muscle samples. By contrasting the muscle transcriptomes of the Piedmontese and Wagyu crosses across 10 developmental time points, our aim was to establish the question to which myostatin is the answer. In other words, what question do we need to ask of the gene expression data for it to reveal the identity of the transcriptional regulator containing the causal mutation?

## Results

### Conventional differential expression

**Figure 1:**
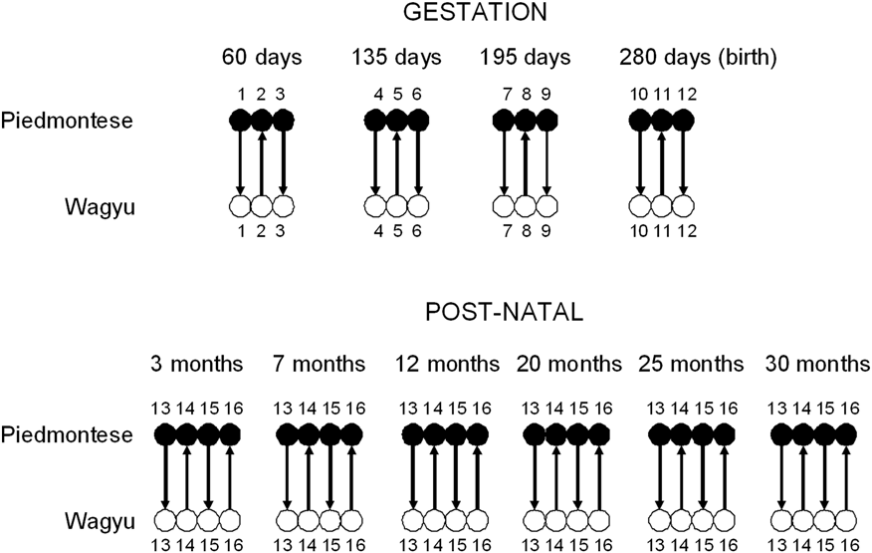
The design of the microarray experiment. Within each cross symbols with the same number indicate samples derived from the same individual animal.

**Figure 2:**
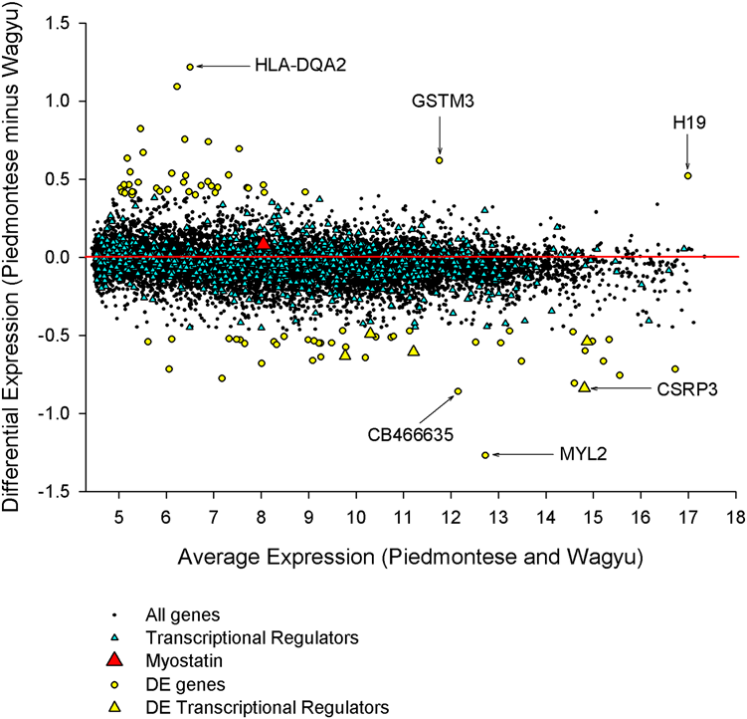
MA plot. Genes expressed more highly in the Wagyu cross are on the bottom, and genes expressed more highly in the Piedmontese cross are on the top. Regulators are denoted by triangles. MYL2 (slow twitch muscle structural protein) is the most differentially expressed gene. CSRP3 is the most differentially expressed regulator. Myostatin is neither abundant nor differentially expressed.

We found that 11,057 genes gave valid expression signal: noise data across the 10 developmental time points for the 2 crosses (Table S1). Of these 11,057 genes 920 were deemed to be gene expression regulators (Table S2). The experimental design (Figure 1) allowed us to assess DE between the crosses and we visualised the data on an MA plot (Figure 2), identifying 85 DE genes using conservative statistical criteria. The most DE genes included slow twitch muscle structural genes (e.g. MYL2), which were higher in the Wagyu crosses (W×H) than in the Piedmontese crosses (P×H) and immune genes (e.g. HLA-DQA2), which were higher in the P×H than in W×H. The most DE transcriptional regulator was CSRP3 which was higher in W×H than in P×H. Consistent with previously published data using a cDNA-based microarray [14] myostatin was not DE between the crosses.

### Differential wiring

Next, we examined the difference in the specific behaviour or co-expression of targeted pairs of genes between the two crosses, by subtracting the correlation coefficient in Wagyu from that in Piedmontese. This approach has a very recent precedent [16]. However, two important modifications presented in the current co-expression work include an absence of significance analysis and the decision to limit the computation to a targeted subset of genes (i.e., transcriptional regulators versus DE genes). This targeting better emphasises the transcriptional regulation of the change in two systems. This quantification of a gene's differential connectivity is more sensitive than the majority of published approaches where only the total number of ‘significant’ connections is contrasted. Instead, like [16] we exploit the identity of the connectors and the differential magnitude of each connection, even in circumstances where the correlation is weak in either (or both) of the networks. As this principal forms the basis of the rest of our analysis and appears to capture the regulatory rewiring that takes place in myostatin mutant muscle, it will be referred to from this point on as differential wiring (DW).

In circumstances where we do analyse changes in total numbers of ‘significant’ connections, we elected to use the term differential hubbing (DH) on the grounds that the total number of connections determines the extent to which a gene can be considered a hub. The PCIT algorithm was used to establish significance in these cases [17]. Table 1 contains definitions for the main terms used in our new analysis, and identifies those aspects which are completely novel (PIF and RIF) from those which have been published in some form (DE, DH and DW).

**Table 1:** The definitions of the terms used in this analysis.

Our Nomenclature	Existing Literature Nomenclature	Formal Definition	Purpose and Further Notes
Differential Expression (DE)	Differential Expression (DE)	The difference between the expression level of a given gene in state 1 minus its expression in state 2	Compares the transcriptional status of a given gene to itself in two states. In longitudinal experiments where DE is averaged across the developmental time points, it will yield a conservative measure of true DE.
Differential Hubbing (DH)	Differential Connectivity (DC)	The difference in the number of significant connections a gene has in two different states e.g. a gene that has 5 significant connections in state 1 and 3 significant connections in state 2 yields a DH of 5−3 = 2	In order to compute differences in the number of significant connections, one first computes which of the co-expression arrangements are significant in the two states. Typically, most connections will be deemed non-significant. The difference between the two states can be computed by subtracting the significant connections a gene has in state 2 from state 1. In the present data this approach fails to identify myostatin as being differentially behaved in Piedmontese versus Wagyu muscle.
Differential Wiring (DW)	Differentially Correlated [16]	The difference in co-expression between a specified pair of genes in two different states. For example GDF8 and MYL2 have a co-expression of +0.761 in the Piedmontese and −0.342 in the Wagyu giving a DW of + 0.761 - - 0.342 = 1.103	This approach forms the basis of our RIF analysis (in conjunction with PIF, see below). In contrast to conventional analyses, no significance testing is used to establish connections.
Phenotypic Impact Factor (PIF)	None, no precedent for the method	The average expression (state 1 and state 2 combined) multiplied by the DE (see above for definition), computed for all DE genes.	A mathematical abstraction quantifying the contribution the various DE genes make to the difference in the molecular anatomy of the two systems. Abundant highly DE genes are emphasised. In the present dataset this enriches for slow muscle structural proteins, correctly reflecting the fibre type shift observed at the gross anatomical level.
Regulatory Impact Factor (RIF)	None, no precedent for the method	The cumulative DW of each regulator relative to the target DE genes, weighted for PIF.	Regulators that are highly DW to the high PIF (i.e., abundant highly DE genes) score highly. In our data, the regulator awarded the highest RIF was myostatin, the causal Piedmontese mutation.

**Figure 3:**
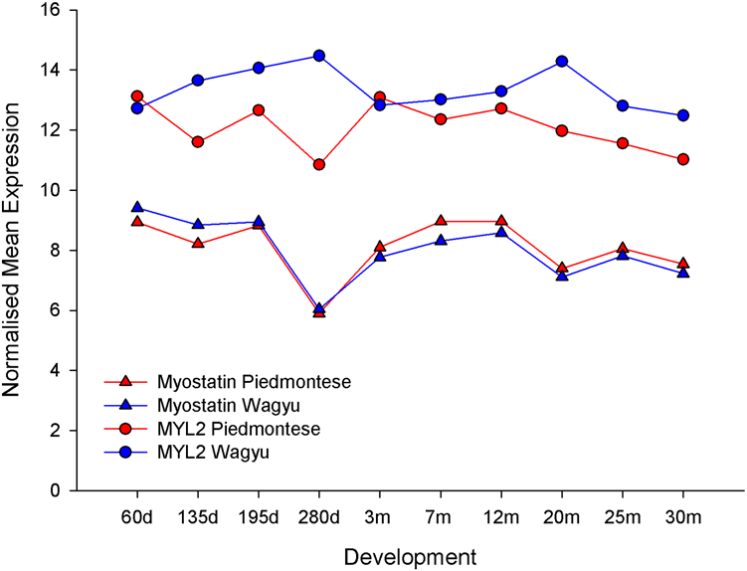
Expression profiles of myostatin and MYL2 in Piedmontese and Wagyu crosses. Myostatin is not differentially expressed, but it is highly differentially wired to the highly DE MYL2.

The most DE gene in our dataset is MYL2, and myostatin is the third most DW regulator to it, with a value of 1.103. The derivation of DW is illustrated for the myostatin-MYL2 connection in Figure 3. It is built on the differences in the myostatin-MYL2 co-expression patterns across development in the Piedmontese cross minus the Wagyu cross. A positive DW is generated where the expression of the target (e.g. MYL2) is positively correlated with the regulator (e.g. myostatin) in P×H, while in W×H the expression is either less positive or negative. The converse applies for negative DW.

### Phenotypic and regulatory impact factors

In an attempt to assess the importance of each DE gene to the change in phenotype, we propose a new metric: the “phenotypic impact factor (PIF).” PIF is a mathematical abstraction designed to ‘weight’ for the contribution the various DE genes make to the difference in the molecular anatomy of the two systems, based purely on their numerical properties. The values were generated by combining the amount of DE between the crosses, coupled with the average abundance calculated for both crosses at all time points for each of the 85 DE genes. Abundant transcripts that were highly DE scored highly, whereas scarce transcripts that were only slightly DE scored poorly. The high phenotypic impact genes enriched for slow twitch muscle structural genes (MYL2, MYL3, TNNT1, MYH7, ACTN2 and MYOZ2) correctly highlighting the observed phenotype change between the breed crosses, namely the gross muscle fibre transition. The coherence of the output is very consistent with an expectation based on the observed gross anatomical fibre change [18].

We formalised this observation using the GOrilla tool [19] comparing the GO terms enriched by high DE to those enriched by high PIF, computed for all 11,057 genes. Not surprisingly, the extremes of both lists strongly enrich for muscle structural components because the transcriptome data was derived from muscle tissue. However, GOrilla assigned a p-value for ‘contractile fibre part’ – the top match within the ‘cellular component’ context - that was 7 orders of magnitude, or 10,000,000 times more significant for extreme PIF than for extreme DE (p = 3.14E-21 versus p = 2.01E-14). We thus conclude that PIF performs well at enriching those genes which appear to contribute strongly to the difference in phenotype between the two states, although a full justification of this conclusion requires further experimental evidence.

On the other hand, the PIF metric is not particularly well suited to regulators, although they were included in the analysis. Regulators are often stably expressed at close to baseline levels making detection of isolated changes in expression level challenging and possibly misleading. To account for this, we ascribed “regulatory impact factors” (RIFs) to each of the 920 regulators based on their cumulative, simultaneous, DW to the DE genes, accounting for the PIF of the DE genes. This metric was intended as a mathematical abstraction to represent the relative importance of the regulators in driving the phenotypically relevant part of the network described above, based on differences in their correlations.

**Figure 4:**
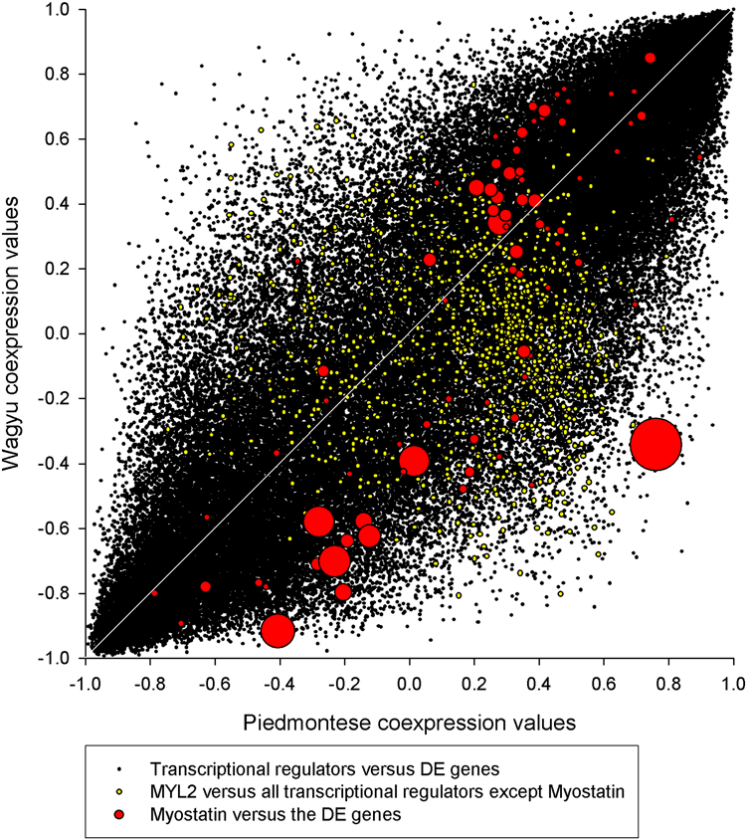
The co-expression relationships between the 920 transcriptional regulators and the 85 DE genes. The red circles represent the co-expression relationships of myostatin to the 85 DE genes, with circle size corresponding to the PIF of the DE gene represented at that particular co-expression intersection (DW). Myostatin is highly DW (as represented by long perpendicular distances from the diagonal) to the highest PIF genes (largest red circles). This dynamic underpins myostatin's exceptional RIF. The density of all points is highest at the extreme co-expression range (i.e., +1, +1 and −1, −1) and lowest for a complete reversal (i.e., +1, −1 and −1, +1).

Those regulators that were highly DW to many of the high PIF genes received strong scores, whereas those that were DW to a few, low PIF genes scored poorly. Figure 4 illustrates the extent to which myostatin is highly DW to the high PIF genes, with Piedmontese and Wagyu co-expressions plotted on the two axes. The 85 red circles correspond to the 85 myostatin-DE gene co-expression values. Circle size corresponds to the PIF of the DE gene co-expressed with myostatin at that particular co-expression intersection. The perpendicular distance from the diagonal line corresponds to the amount of differential wiring. For myostatin, this distance tends to be greatest for the high PIF genes (largest circles). The five largest circles are MYL2, CSRP3, MYH6, CA3 and MYL3.

It is important to note that Figure 4 also reveals that most of the mass (i.e., most of the correlation coefficients) are either close to −1 or close to +1. This bimodal distribution in the correlation coefficients affecting DE genes has already been documented [20] and contrasts with the expected uni-modal distribution that would be obtained across all genes where most of the mass is centred around zero.

We explored 2 alternative methods to compute RIF scores (Eq4 and Eq5 Materials and Methods). Myostatin had the fourth most positive RIF using Eq4 and the second highest using Eq5 (Table 2). Overall the RIF values calculated using the two equations had a correlation efficient of ∼0.7.

**Table 2:** The top 10 positive differentially wired regulators P×H v W×H.

Regulator	DE	Gene Function	RIF	Rank Eq4	Rank Eq5
MSTN	no	Causal mutation in double-muscled Piedmontese cattle, negative regulator of muscle mass. TGF-ß signalling.	3.49	4	2
MEF2C	no	Muscle transcription factor	3.21	37	1
SUV39H2	no	Histone methyltransferase. Cooperates with SMADS to repress promoter activity. TGF-ß signalling.	3.13	3	4
ACTL6B	no	Regulation of genes in the brain	3.02	14	5
HNRNPD	−0.41	Pre mRNA processing	3.01	10	6
MYOD1	−0.41	Master regulator of muscle cell differentiation	2.94	58	3
ATRX	no	Chromatin remodelling	2.85	106	7
IRF9	no	Interferon regulatory factor	2.82	67	9
CCNK	no	Regulation of transcription	2.79	160	8
HAT1	no	Histone acetyl transferase	2.79	13	11

**Figure 5:**
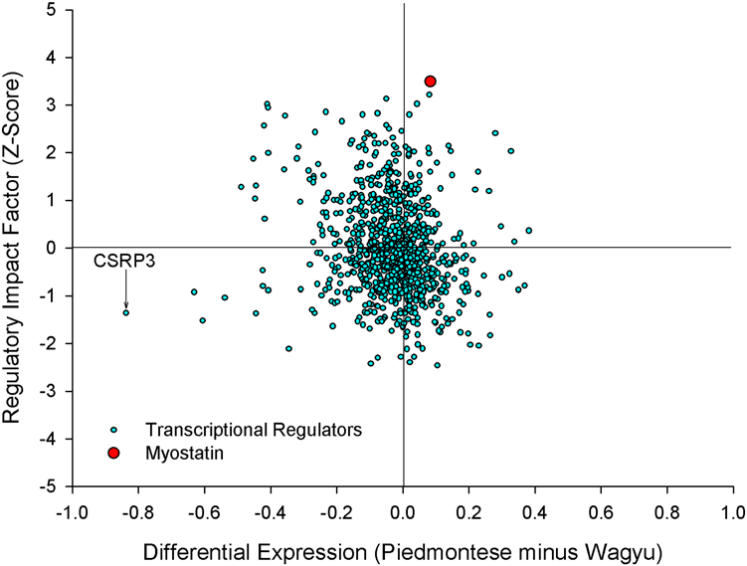
The relationship between regulatory impact factor and differential expression. The DEs of the 920 regulators are plotted against their respective RIFs (mean dot Eq4+Eq5). Myostatin, indicated by a red dot, is awarded the highest RIF despite not being DE.

In the absence of evidence favouring one approach over the other we decided to follow the original thread of defining the question to which myostatin was the answer. When we calculated the mean of the two different RIF values, myostatin received the highest score out of the 920 regulators with a RIF of 3.49 (Figure 5 and Table 2). Two muscle transcription factors MEF2C and MYOD1 also appeared in the top ten, although the former was ranked much lower by Eq4. In addition, SUV39H2 (a histone methyltransferase that cooperates with SMADs [21], components of the TGF-ß pathway though which myostatin is proposed to act, lay in third place (Table 2). No major muscle TF, or components of the TGF-ß pathway, were in the top ten negative RIF genes (Table 3). The remainder of the top 10 positive and negative RIF regulators are annotated in Tables 2 and 3, and can be compared and contrasted to the top 10 positive and negative DE regulators in Tables 4 and 5.

**Table 3:** The top 10 negative differentially wired regulators P×H v W×H.

Regulator	DE	Gene Function	RIF	Rank Eq4	Rank Eq5
HOXB13	no	Body patterning along main axis, suppressor of cell growth	−2.46	906	920
IFRD1	no	Interferon-related development regulator 1	−2.42	882	919
CDK7	no	Link between regulation of transcription and cell cycle	−2.39	885	918
FOSL2	no	Regulator of cell proliferation, differentiation and transformation	−2.30	898	917
MYT1	no	Myelin transcription factor	−2.28	914	915
MAFK	no	Erythroid transcription factor	−2.28	870	916
PADI4	no	Possible role in granulocyte and macrophage development	−2.12	853	914
LMCD1	−0.34	Negative regulator of muscle cell differentiation	−2.11	874	913
CTNND2	no	Catenin delta 2	−2.10	897	910
KLF15	no	Kruppel-like factor 15	−2.05	857	912

**Table 4:** The top ten positively differentially expressed regulators P×H v W×H.

Regulator	DE	Gene Function	RIF	Rank Eq4	Rank Eq5
HOXB6	0.37	Regulation of development	−0.80	869	718
TFDP2	0.35	Transcription factor, E2F dimerization partner 2	−0.88	476	764
HOXB5	0.34	Regulation of development	0.13	286	352
BHLHB5	0.33	Brain transcription factor	2.03	91	40
FOXO1	0.32	May play a role in myogenic growth and differentiation	−0.54	481	637
SCAND1	0.30	Peroxisome proliferative activated receptor, gamma, coactivator 1, role in lipid metabolism	−0.62	664	666
BACH2	0.30	B-cell leucine zipper transcription factor	0.45	245	254
MLLT10	0.28	Remodelling histones/nucleosomes	2.40	101	17
FOXQ1	0.26	TGFB2 pathway	−1.82	1	39
MAX	0.26	Role in cell proliferation and differentiation	−1.39	162	50

**Table 5:** The top ten negatively differentially expressed regulators P×H v W×H.

Regulator	DE	Gene Function	RIF	Rank Eq4	Rank Eq5
CSRP3	−0.83	Positive regulator of myogenesis	−1.36	883	862
BTG2	−0.63	Cell cycle regulator, anti-proliferative	−0.93	701	795
ATF3	−0.60	Negative regulator of Toll-like receptor 4	−1.52	860	881
ANKRD1	−0.53	Positive regulator of myogenesis	−1.04	429	817
CDK9	−0.49	Cell cycle regulator	1.28	531	97
FOS	−0.44	Cell differentiation and proliferation in bone, cartilage and blood TGF-ß signalling	−1.37	826	867
CILP	−0.44	Negatively regulates TGF-ß signalling	1.30	7	123
FST	−0.42	Positive regulator of muscle mass TGF-ß signalling	−0.47	575	602
HOMER2	−0.42	Negative regulator T cell activation	−0.80	339	729
FRZB	−0.41	Negative regulation of Wnt signalling	2.56	826	907

To highlight which cluster of DE genes are being ‘perturbed’ by which cluster of regulators, the DW values for the 920 regulators (in rows) and the 85 DE genes (in columns) (Table S3) can be assembled into a ‘perturbation matrix’ which we visualised using PermutMatrix software [22]. This novel representation of gene expression data (derived from the more traditional configuration with genes in rows and samples in columns) allows for the separation of DE genes from regulators and, after hierarchical clustering, reflects the way in which the regulators are co-differentially wired with each other (i.e., where the differential wiring behaves in a co-ordinated manner).

**Figure 6:**
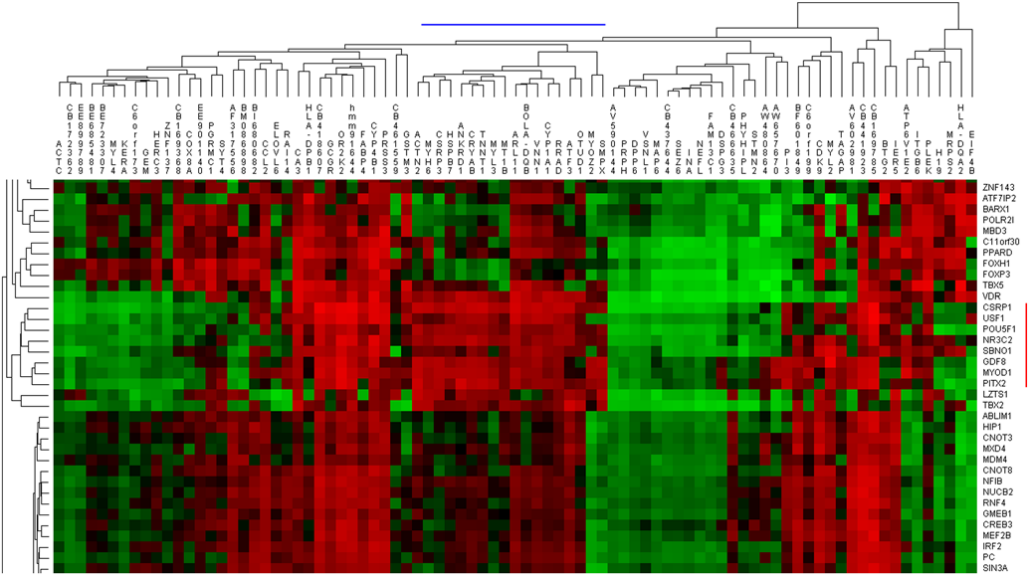
The P×H v W×H “perturbation matrix.” We applied Permut Matrix's hierarchical clustering algorithm to both rows (920 regulators) and columns (85 DE genes). A subset of the full matrix including the high phenotypic impact slow twitch module (blue line) and the major high impact transcriptional regulator circuit (red line). The scale is −1.53 (bright green) to +1.53 (bright red), with 0 being black.

In Figure 6 a small section of the perturbation matrix is shown. Of particular note was a tight cluster of 18 DE genes comprising 5 genes encoding high PIF slow twitch structural proteins (MYL3, TNNT1, MYH7, ACTN2 and MYOZ2) and also featuring SMPX and 2 DE regulators (ANKRD1 and CSRP3). MYL2, another gene encoding a slow twitch structural protein, did not feature in this DE module, but clustered on its own. The regulatory axis contained several high impact regulatory ‘hot spots.’ One of these included myostatin and MYOD1 at its heart, and also included CSRP1, USF1, POU5F1, NR3C2, SBNO1 and PITX2. The very tight clustering of myostatin and MYOD1 reflects closely coordinated patterns of DW between the two crosses across the 85 DE genes.

**Figure 7:**
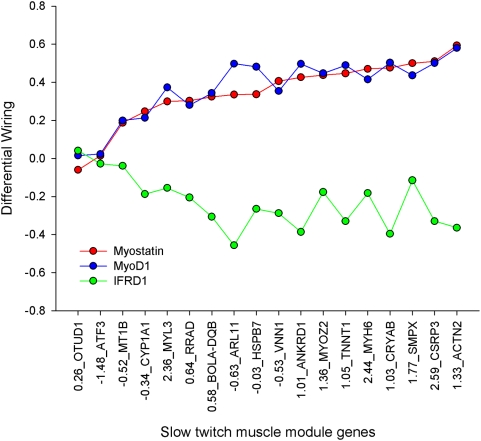
The basis of an edge in a co-differential wiring network. Myostatin, MyoD1 and IFRD1 are highly co-differentially wired across the 85 DE genes (correlation coefficients >0.9 or <−0.9). Here their respective relationships are visualised against only those 18 DE genes that cluster into the slow twitch Permut module, but the relationship holds for all 85 DE genes.

These biologically-sensible clusters imply that co-differential wiring can be used as an explicit criterion to form an edge in a regulatory perturbation network. We used a hard 0.9 threshold to establish network edges between those regulators that were highly co-differentially wired to the 85 DE genes. We visualised the deduced network in Cytoscape [23]. This approach led to an enormous cohesive module of low impact regulators (those regulators that apparently do not contribute to the change in phenotype), plus a number of smaller high impact modules (those regulators that apparently do contribute to the change in phenotype). A notable high impact module comprised 3 transcriptional regulators: MSTN, MYOD1 and IFRD1. The derivation of the high co-differential wiring between the crosses for myostatin and MYOD1 is illustrated in more detail with specific respect to the slow twitch module genes in Figure 7. In contrast to myostatin and MYOD1, which are highly positively co-differentially wired to each other, the other member of the module, IFRD1, is highly negatively co-differentially wired to them. The greatest DW values for all three transcriptional regulators tend to be associated with the high PIF muscle structural genes at the far right of the x axis (ANKRD1, MYOZ2, TNNT1, MYH6, SMPX, CSRP3 and ACTN2).

However, positive correlation of DW of regulators does not necessarily imply positive correlation, or indeed any significant correlation, of expression of the regulators themselves and vice versa. In other words, neither the clustered regulators on the y axis of the perturbation matrix nor the clustered DE genes on the x axis are actually significantly co-expressed with each other in any combination, based on a PCIT analysis (unpublished data). Furthermore, Myostatin, MyoD1 and IFRD1 are not significantly co-expressed with any of the other 11,057 genes in the system, let alone the subset in the matrix. The same applies to ACTN2, MYH6, CSRP3, ANKRD1, MYL3 and MYOZ2 (unpublished data). Rather, it is the coordinated manner in which two genes *differ* in their behaviour in the two systems that drives co-differential wiring.

### Data simulation

We tested the distributional and numerical properties of RIF1 and RIF2 (Eq4 and Eq5) on a simulated data to assess the extent to which our real output could be ascribed to chance. The simulated data comprised 5,000 genes surveyed across 10 experimental conditions (in line with the 10 time points) in two treatments (in line with the two breed crosses). In accordance with the real data, expression values were simulated from a normal distribution with a mean of 8.6 and a standard deviation of 2.8 and truncated at 4 and 16. Also, for each gene, its expression profile across the two treatments was simulated to have a correlation of 0.95.

Simulations were performed under the null hypothesis of no differential expression between treatments, no correlation between genes across conditions, and no regulator-target relationships. Therefore, in these settings any observed association could be attributed to chance alone.

For the computations of RIF1 and RIF2, a random 920 genes were selected and treated as potential regulators and their regulatory impact factor computed against the 85 genes showing the most extreme measure of differential expression across the two conditions. Based on this approach a simulated version of Figure 4 was constructed (data not shown) which, unlike the observed Figure 4 from our real data, bore most of its mass in its centre (indicative of a bell-shaped distribution of correlation coefficients). Both distributions were found to be statistically different as indicated by the Kolmogorov-Smirnov two-sample test (P<0.0001).

### Differential hubbing

**Figure 8:**
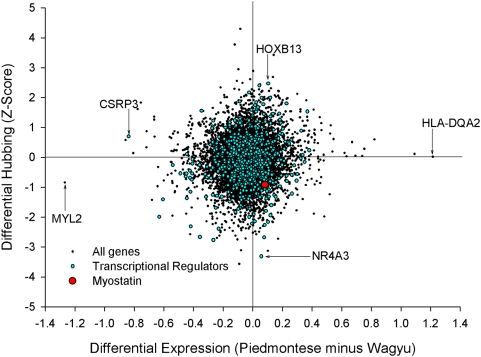
The DE and differential hubbing of all 11,057 genes. While the extremes of the DH axis enriched for transcriptional regulators in general, myostatin is neither DH nor DE.

We used the PCIT algorithm [17] to establish the number of significant connections for each regulator in the P×H and W×H datasets, to determine how well this conventional approach performed in comparison to RIF. In line with previous authors we discovered that the DH axis (i.e., the change in the number of significant connections between the two breeds) enriches at its extremes for transcriptional regulators (Figure 8). The extreme 1% DH (i.e., 110 genes out of the 11,057 available) contains 15 transcriptional regulators rather than the 9 expected by chance alone (hypergeometric p-value = 0.0192). This enrichment is not true for the DE axis, which contains 9 transcriptional regulators. However, DH failed to capture myostatin in its extremes, which suggests its usefulness as a metric for the identification of transcriptional regulators of relevance may not be broadly applicable (Figure 8). We also ran the ‘signed’ hubbing analysis of [24] on our data and plotted the output (Figure S1). As with the PCIT DH approach, myostatin was not enriched at the extremes of the DiffK DH axis. This means it failed to identify the regulator containing the known causal mutation as being differentially behaved in the two muscle systems.

## Discussion

### Reducing type III error by defining a cogent question

In the introduction we posed a computational challenge: identify the question in P×H versus W×H muscle development to which myostatin is the answer. The subsequent analysis suggests the following: “Which transcriptional regulator is cumulatively most differentially wired to the abundant most differentially expressed genes?” This question is clearly very different to the conventional “which transcriptional regulator is the most differentially expressed?” and unsurprisingly the latter gives quite different answers, including the notable failure to identify myostatin out of the 920 candidates.

This result suggests that traditional microarray approaches generating lists of DE regulators may be committing type III statistical error, the error committed when giving the right answers to the wrong questions [25],[26]. Regulators may indeed be correctly identified as DE, but this does not mean that they are differentially activated. The converse is also true. For example, TF activity can be regulated in many ways, localisation to the nucleus, chemical modification, change in accessibility of DNA binding sites and availability of cofactors that by and large are independent of TF expression level. It is therefore inevitable that these common forms of regulatory change will be overlooked by DE analysis.

The positive identification of myostatin as the major regulatory perturbation in this specific set of experimental contrasts is noteworthy, despite the stated aims of the approach. The Piedmontese causative mutation exists at the first level of organisation (genomic DNA), and manifests its effect at the third (protein) and higher levels (phenotype). Despite this, we can identify it using only data at the second level of biological organisation – the transcriptome. In addition, all animals were Hereford hybrids so 50% of the protein translated by the P×H animals was as functional as the myostatin protein translated by the W×H; in line with this, the increase in muscle mass was correspondingly subtle (∼9%) (unpublished data).

### Assigning phenotypic and regulatory impact factors

The new algorithm works, in effect, by firstly establishing a Phenotypic Impact Factor (PIF) for each of the DE genes. Thus, genes that are both highly abundant and highly DE between the crosses derive a correspondingly high PIF, or discrimination factor. Taken together, this weighting provides an abstract molecular description of the phenotype perturbation specific to the treatments under consideration. In the P×H versus W×H comparison, the genes with the highest PIF (i.e., those that are abundant and highly DE) tend to be slow twitch muscle structural genes (MYL2, MYL3, TNNT1, MYH7, ACTN2 and MYOZ2). This correctly reflects the most pervasive phenotypic change in Piedmontese myostatin mutants (along with the increase in muscle mass) namely the gross fibre type transition. We therefore conclude that DE, in the context of transcript abundance, is a powerful measure of phenotypic / anatomical change (but not necessarily, as we have already argued, regulatory change).

RIF is based on the cumulative, simultaneous, differential wiring (DW) of each regulator to the DE genes, ‘weighted’ for the PIF of each DE gene. Satisfactorily, the regulator awarded the highest RIF by this approach is myostatin, the gene that bears the known causal mutation (SNP) in Piedmontese genomic DNA [11]. This positive result suggests that our concept and method of assigning RIF represents a promising approach to the identification of causal mutations, and additionally those regulatory ‘hot spots’ resulting from non-genetic perturbations in other systems.

The highest impact regulators are documented in Table 2, and include known muscle master regulators like MyoD1. A caveat: some known muscle master regulators (e.g. Myf5) do not perform strongly in our analysis, i.e., they are ascribed relatively low RIF's. This suggests not that these regulators are unimportant to bovine muscle development, but rather that they play only a small role in the rewiring that directs these two muscle phenotypes down different developmental paths.

During the conceptual development of the algorithm we tried several permutations. The best performer, as described above and in the results section, incorporates the average abundance and differential expression of the DE genes (which tend not to be transcriptional regulators), and the cumulative DW of the regulators to those weighted DE genes. Surprisingly, inclusion of either the average abundance or DE of the regulators themselves actually impairs the ability of the algorithm to identify myostatin (data not shown).

While we assessed several versions of the algorithm, there is no evidence that the data has been over-fitted because (1) the model is relatively simple compared to the data it analyses, (2) like in any other expression experiment, only the normalized gene expression levels for each gene in each of the samples (or experimental conditions) are needed, (3) it is built on sound mathematical principles (mixed-ANOVA models and model-based clustering), and (4) those mathematical principles mesh well with our biological understanding of the behaviour of both structural proteins (where DE and abundance are always important) and transcriptional regulators (where DE and abundance are not necessarily important, but transcriptional connectivity is important) in a range of living systems.

The two versions of the algorithm provided (Eq4 and Eq5) are alternatives in the sense that they are built on the same set of concepts. However, at this stage, it is not clear whether one can be considered superior to the other. Consequently, we have derived our impact factor discussion from the combined, averaged output of both equations (Table S2 and Tables 2 and 3). Equation 5 has the appealing intrinsic mathematical feature that it allows for auto-regulation, a biological feature thought to be true for myostatin itself [11].

### Conventional differential expression

Our observations imply caution when assessing isolated DE lists of TF. That TF can behave differently in two systems without being strongly DE, has been discussed before [24],[27] and is graphed for these data in Figures 2, 5, and 8. It is interesting that the top 10 candidates generated by the new algorithm and the top 10 DE regulators do not overlap, although HNRNPD and MYOD1 lie just outside the top ten most DE regulators; the most DE regulator (CSRP3) was assigned only a modest RIF. CSRP3 has been reported to be a potential structural component of the sarcomere [28], but also as a soluble component [29] and as a TF involved in the transduction of mechanical stress signals from the structural proteins to the nucleus [30],[31]. ANKRD1, another major DE regulator, may have a similar role [32],[33]. The possible structural roles of these regulators may place them in an intermediate category between structural protein and regulator, which complicates the decision to include them in a transcriptional regulator list and may also have implications for interpretation of the output.

### Wiring the regulatory network

Assigning impact factors to the regulators (based on the behaviour of its co-expression with respect to the phenotypically most relevant part of the network) forms step 1 in a 2-step process, and it yields biologically valid results. The next step is to computationally wire up the high impact regulators into coherent transcriptional modules, whose coordinated behaviour drives the phenotype change. We attempted to do this by establishing relationships between regulators who were ‘similarly’ or co-ordinately differentially-wired between the two crosses. To our knowledge this is the first time co-differential wiring has been used for reverse-engineering regulatory circuitry. The resultant output captures the phenotypic and regulatory differences between the two crosses and so we view it as a ‘perturbation matrix.’

The building and clustering of the perturbation matrix satisfactorily resolves both axes into biologically sensible modules. For example the DE axis generates a very tight module of high phenotypic impact slow twitch muscle fibres (ACTN2, MYH7, TNNT1, MYL3 and MYOZ2). Equally, the regulator axis resolves a high impact regulatory module comprising myostatin and MYOD1, among others. Myostatin is embedded in the middle of this high impact module. We interpret these clusters of regulatory disturbance as representing ‘hot spots’ of circuit rewiring that account for the major phenotypic changes between the crosses.

The exceptionally tight coupling of myostatin and MYOD1 on the y axis is the product of a near perfect matching of co-differential wiring across all 85 DE genes (p = 0.917). Figure 7 illustrates the co-differential wiring of these two regulators against the slow twitch module DE genes. Unfortunately, our success in correctly determining the rewiring of myostatin in (i.e., identifying the other regulators through whom it communicates its effect on muscle mass and muscle fibre composition) is harder to evaluate than the impact factor data. This is because the regulatory events that transduce myostatin's influence on Piedmontese muscle mass and fibre composition have not been well established. To establish the validity of the co-differential wiring approach we examined the biological identity of those genes with the highest co-DW coefficients. The distinction belongs to PTTG1 and TOP2A (0.994) which are involved in the same highly fundamental biological process, that of chromatid separation during DNA replication.

With specific regard to the myostatin and MyoD1 clustering, the high co-DW congruence makes a clear prediction that the myostatin SNP in Piedmontese exerts its effect on skeletal muscle via circuit rewiring with MyoD1. MyoD1 has not only been shown to drive the expression of a set of genes necessary for fast muscle differentiation [34], but also to be specifically regulated by myostatin in mice [35]; thus, our prediction appears robust. The separation of abscissa clustering predicts that MYL2 is under a different regulatory program to the other slow twitch muscle structural genes in the system.

When we next used the co-DW patterns to generate edges in a network, myostatin was linked to 2 other high impact regulators, MYOD1 and IFRD1. It is highly noteworthy that IFRD1, which is required for myoblast differentiation, forms a known, experimentally-verified regulatory circuit with MYOD1 [36] adding further support to our co-differential wiring method. Taken together, these results are very appealing because they indicate a single method that not only correctly clusters regulators who behave the same in the two systems (PTTG1 and TOP2A) but also those who behave differently in the two systems (Myostatin, MyoD1 and IFRD1). The additional myostatin module connections, i.e., between myostatin and IFRD1 (−0.903) and between MYOD1 and IFRD1 (−0.925) (Figure 7) are not represented in Figure 6 because Permut Matrix does not recognise inverted patterns (i.e., the signs on the edges are negative instead of positive). In contrast to MYOD1, the high RIF MEF2C is not part of this cluster.

### Qualifiers

The utility of this algorithm clearly relies on appropriate data selection. Presumably, the microarray data must be assayed on the right tissue and at biologically important times. However, the dataset that we analysed was not designed to address the specific question of identifying the gene containing the causal mutation, rather it was designed to study the impact of nutrition restriction of the mother on the subsequent performance of the calves [37],[38]. A limitation of our method is that the regulators must be identified at the start of the analysis. However, other than the initial identification of the regulators, all the downstream information such as PIF, RIF and the topology of the co-differentially wired network is entirely data-driven, i.e., computed directly from the normalised microarray expression values.

Finally, during the development of the algorithm we initially attempted to determine regulatory changes via a simpler version of connectivity, i.e., describing changes in the *number* of connections of each regulator, what we have termed DH (and what is sometimes referred to as differential connectivity in the literature). DH approaches have previously proved useful in identifying genes that appear to play key regulatory roles in evolution [39], cancer [40] and the development of sexual dimorphism [41]. However, the procedure is limited because it requires the application of a significance analysis to isolate the significant from the non-significant connections. Which significance analysis to use is a subject of ongoing debate with weighted networks appearing to hold the most promise [17],[24],[42],[43].

While it was true that high DH (coupled with low DE) proved diagnostic of regulators in general (*sensu* [24],[27]), it performed poorly as a discriminatory metric with specific regard to myostatin (Figure 8). A possible reason why can be illustrated by the following hypothetical example. Consider a regulator with 100 positive associations in one system, and 100 negative associations in another, and two entirely different sets of target genes. In its most basic form, a DH analysis would suggest this regulator is not differentially hubbed (as 100−100 = 0), clearly a false negative. Thus, a DH analysis may suffer from (1) ignoring the identity of the connected genes, (2) ignoring the sign on the edges, and (3) ignoring the phenotypic impact of each connected gene. To further compare RIF to published network approaches, we ran the DiffK hubbing analysis of [24], which is a sophisticated ‘signed’ differential hubbing algorithm. This positioned myostatin 147^th^ out of the 920 regulators on the DiffK axis, i.e., it failed to identify myostatin as behaving differently in the two muscle systems (Figure S1). This result suggests that hubbing analyses, in their various forms, are unable to identify the causal mutation in this particular data.

Our definition of RIF does not require computation of the number of connections of a given regulator in each of the two networks. Therefore, algorithms for network re-construction (weighted or otherwise) are of no relevance. Instead, the difference between the connection weight of a given gene with each of the DE genes, accounting for PIF, appears to be sufficient. In other words, RIF has a set of refinements which make it highly sensitive. These refinements include recognising the specific identity of target genes, recognising the possible importance of ‘weak’ edges that would be deemed non-significant by other methods and recognising the phenotypic importance of the target genes.

This principle is well illustrated by the DW of myostatin to MYL2. The co-expression relationship significantly changes from +0.761 in the P×H system to −0.342 in the W×H system. The −0.342 Myostatin-MYL2 ‘edge’ in the Wagyu network would be unequivocally discarded by all statistical methods as being insignificant (whether by ARACNE, PCIT or some other approach) whilst the +0.761 Myostatin-MYL2 ‘edge’ in the Piedmontese would be borderline insignificant depending on the exact analysis used. Therefore, comparisons between these arrangements (which underpin the success of our present analysis) cannot be sensitively quantified by DH. Further, the fact that MYL2 is highly abundant and highly DE (and therefore of great phenotypic importance) would be overlooked by DH, unless the PIF metric was applied. It is a telling observation that myostatin is neither DE nor DH (Figure 8), yet is cumulatively the highest RIF regulator on the array by some margin (Figure 5).

### Conclusions and future directions

We have argued that the algorithms success is built on controlling type III error, i.e., it gives the right answer because it asks the right question. The approach should be generalisable to other ‘omics data because its mathematical approaches mesh well with the known biology of regulatory and non-regulatory molecules. Unlike other causal mutation finding computational approaches [6]–[9], RIF requires data at only one level of organisation (the transcriptome). Having said this, the future availability of more complete TF binding data and other resources will enable the determination of a more complete path from causal mutation to phenotype. By extracting richer regulatory information RIF may help establish novel regulatory perturbations. These include a better understanding of the network topologies that underpin evolutionary novelty and the mis-wiring events that lead to aberrant development such as cancer.

## Materials and Methods

### Ethics statement

Use of animals and the procedures performed in this study was approved by the New South Wales North Coast Animal Care and Ethics Committee (Approval No. G2000/05).

### Animals and samples

Hereford cows were artificially inseminated or mated to one of 5 different Wagyu sires or one of 6 different Piedmontese sires. All Piedmontese sires were homozygous for the MSTN (GDF8) missense mutation in exon 3 and none of the Wagyu sires carried the mutation. We sequenced the myostatin transcript from cDNA and found it to be heterozygous for the SNP mutation in all Piedmontese samples with approximately equal peak heights for both alleles. Muscle tissue from these animals has been contrasted previously across both pre- [14] and post- [15] natal development using a custom cDNA array derived from adult muscle and adipose tissue libraries. Further details relating to experimental design can be found therein. Total RNA was prepared as previously described [14].

### Microarray platform and experimental design layout

We used a bovine oligonucleotide microarray, developed in 2006 by ViaLactia Bioscience in collaboration with Agilent, containing 21,475 unique 60-mer probes, representing approximately 19,500 distinct bovine genes. Four microarrays are present on each Agilent chip. Issues considered in the experimental design included the availability of biological replicates as well as the quality of the extracted mRNA. The experimental layout was designed to allow a focus on the cross comparison, but to also permit a developmental aspect to be carried out (Figure 1). Two clear components were included: gestation and post-natal. For the gestation component of the experiment, a total of 12 dual-channel hybridizations were performed including three biological replicates for each cross and at the 4 time points (60, 135, 195 and 280 days). For the post-natal component, 36 hybridizations were performed including the same four biological replicates for each cross surveyed at six ages from 3 to 30 months old. Alternate dye channel was applied to allow accounting for systematic effects due to dye bias. Microarrays were hybridized at the SRC Microarray Facility of the Institute for Molecular Biosciences in Brisbane, Australia (http://microarray.imb.uq.edu.au/).

### Generation of the list of regulators

We used a number of approaches to establish a reasonably definitive list of genes encoding proteins that directly or indirectly modify gene expression, including chromatin remodelers. We made use of a comprehensive list of TF previously published in humans [44] and identified the homologs on the Agilent bovine array. This list was augmented by examining files available at ftp://ftp.ncbi.nih.gov/gene/DATA/ which were obtained and searched by accession number to identify gene ontology information for each sequence. We also took advantage of a range of online databases with information on TF binding motifs to further corroborate the list. While we discriminated between modifiers of gene expression, such as TF, non-transcription factor regulators (e.g. myostatin), chromatin remodelers (e.g. HDAC2) and signalling molecules (e.g. FRZB) in the list, the phrase transcriptional regulator covers all together.

### Microarray data processing, normalization and differential expression

Gene expression intensity signals were subjected to a series of data acquisition criteria based on signal to noise ratio and mean to median correlation as detailed previously [45]. In brief, we employed the following two editing criteria for data acquisition: First, we required that the signal to noise ratio (computed by dividing the background corrected intensity by the standard deviation of the background pixels) be greater than unity; Second, we required that the correlation between the mean and the median signal intensities (computed by dividing the smaller of the mean or median by the larger) to be greater than 0.85. Tran et al. [46] suggested that a correlation of 0.85 or higher not only retains more data than other methods, but retained data are more accurate than traditional thresholds or common spot flagging algorithms. However, these criteria were applied separately for the red and for the green intensity channels so that a different number of observations for each channel were obtained. These resulted in a total of 2,083,641 gene expression intensity readings (1,027,379 red and 1,056,262 green) on 11,057 genes that were background corrected and base-2 log transformed. The arithmetic mean and standard deviation (in brackets) for the red and green intensities were 8.67 (3.16) and 8.14 (2.82), respectively.

Data normalization was carried out using a linear mixed ANOVA model as described in [47] and differentially expressed (DE) genes identified by model-based clustering via mixtures of distributions on the normalized expression of each gene at each cross and time point as detailed in [47],[48]. In brief, the following linear mixed-effect model was fitted to the data:
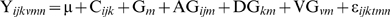
(1)where Y*_ijkvmn_* represents the *n*-th background-adjusted, normalized base-2 log-intensity signal from the *m*-th gene at the *v*-th experimental variety treatment (breed cross and time point) from the *i*-th chip, *j*-th array (i.e., there are four microarrays per chip) and *k*-th dye channel; μ is the overall mean; C represents a comparison fixed group effects defined as those intensity signals from the same chip, array and dye channel; G represent the random gene effects with 11,057 levels; AG, DG, and VG are the random interaction effects of array by gene, dye by gene, and variety by gene, respectively. Finally, ε is the random error term. In what follows, it is understood that the *v*-th variety treatment incorporates both the main class treatment of cross (e.g. P×H versus W×H) as well as the sub-class level (e.g. the 10 time points). That is: *v* = 1, 2, …, 10 for the Piedmontese cross at the 10 time points; and *v* = 11, 12, …, 20 for the Wagyu cross also at the same 10 time points.

For the random effects in model (1), standard stochastic assumptions are:
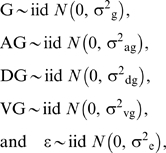
where iid denotes independently and identically distributed and *N* denotes the normal distribution. Variance components are between genes (σ^2^ *_g_*), between genes within array (σ^2^ *_ag_*), between genes within dye (σ^2^ *_dg_*), between genes within treatment (σ^2^ *_vg_*) and within genes (σ^2^ *_e_*). Variance components were estimated using restricted (to zero error contrasts) maximum likelihood (REML; see [49] for detailed formulae).

To determine which genes are DE between the two crosses, the following t-statistic was computed for each gene in *g*:
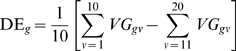
(2)This definition of DE is likely to be conservative as it is based on overall variation in expression across all time points. However, it has the advantage of dealing with irregular time intervals compared with dynamic clustering methods based on autoregressive models [50], where the time points have to be evenly spaced.

Finally, the DE measurement contrasts in (2) were processed by fitting a two-component normal mixture model and posterior probabilities of belonging to the non-null component were used to identify DE genes with an estimated experiment-wise false discovery rate of <1% as described by [51].

### Differential wiring

We introduce the term differential wiring (DW) which, defined for every pair of genes, is computed from the difference between the co-expression correlation observed between these two genes in the Piedmontese network minus the co-expression correlation between the same pair of genes in the Wagyu network.

In algebraical terms, DW is computed as follows:
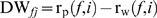
(3)where *f* and *i* indicate the *f*-th TF and the *i*-th DE gene, respectively;

r_p_(*f,i*) is the correlation coefficient between the expression of the *f*-th TF and the *i*-th DE gene in the Piedmontese cross; and
r_w_(*f,i*) is the equivalent for the Wagyu cross.

### Regulatory impact factors

For every regulator in our dataset, we introduce a new term, namely Regulatory Impact Factor (RIF) which simultaneously combines the DW between the TF and each of the DE genes, weighted for the PIF of the DE genes, i.e., their expression averaged across the two crosses (denoted as A*_i_*, for the *i*-th DE genes) and their measure of differential expression given in Equation (2).

In algebraical terms, the RIF associated with the *f*-th TF is computed as follows:
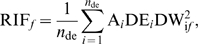
(4)where *n*_de_ denotes the number of DE genes. An alternate definition of RIF*_f_*, providing similar rankings to (6) is given by:
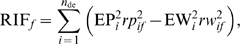
(5)where 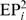 and 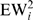 indicate the expression of the *i*-th DE gene in Piedmontese and Wagyu, respectively; and 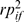 and 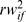 indicate the square of the co-expression correlation between the *f*-th TF and the *i*-th DE gene in the Piedmontese and Wagyu networks, respectively. This alternate definition of RIF has the additional appealing features of expressing the product of the average and the differential expression as the difference of the squared expression in each cross (i.e., a computational simplification), as well as the squared correlations (i.e., coefficient of determination) between the *f*-th TF and the *i*-th DE gene indicating the strength of one variable (the TF) explaining variation in a second variable (the DE gene). It also allows for the existence of self-regulation which more realistically reflects biology (i.e., note that for DW*_fi_* = 0 for *f* = *i*; a situation where a TF is also DE, impacting on the computation of RIF as per Equation 4). RIF scores were normalized to a mean of zero and a standard deviation of one.

PIF is implicit in the Equation 4 representation of RIF and is defined as the product of the average and the differential expression of a gene, computed as follows:
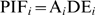
(6)


### Differential hubbing

Differential hubbing was calculated in two ways. Firstly, by subtracting the number of significant connections a gene has in Wagyu from the number of significant connections it has in Piedmontese where significance was established using the PCIT algorithm [17]. Secondly, we also computed a ‘signed’ DH using the network strategy detailed in [24].

## Supporting Information

Figure S1 The DE and DiffK for all 11,057 genes. Myostatin is not DiffK.
(0.28 MB TIF)

Table S1 The normalised mean expression for the 11,057 genes across the ten developmental time points for the two breed crosses.
(4.30 MB XLS)

Table S2 The list of the transcriptional regulators (column 1) with their DE (column 2) and their combined, averaged RIF scores from Eq4 and Eq5 (column 3).
(0.09 MB XLS)

Table S3 The differential wiring arrangements (Piedmontese coexpression minus Wagyu coexpression) for the 920 regulators versus the 85 DE genes.
(1.39 MB XLS)
