# Supplementary figures and images for: A Differential Wiring Analysis of Expression Data Correctly Identifies the Gene Containing the Causal Mutation
Source: PLoS Comput Biol. 2009 May 1;5(5):e1000382. doi: 10.1371/journal.pcbi.1000382 (PMC2671163; doi:10.1371/journal.pcbi.1000382)

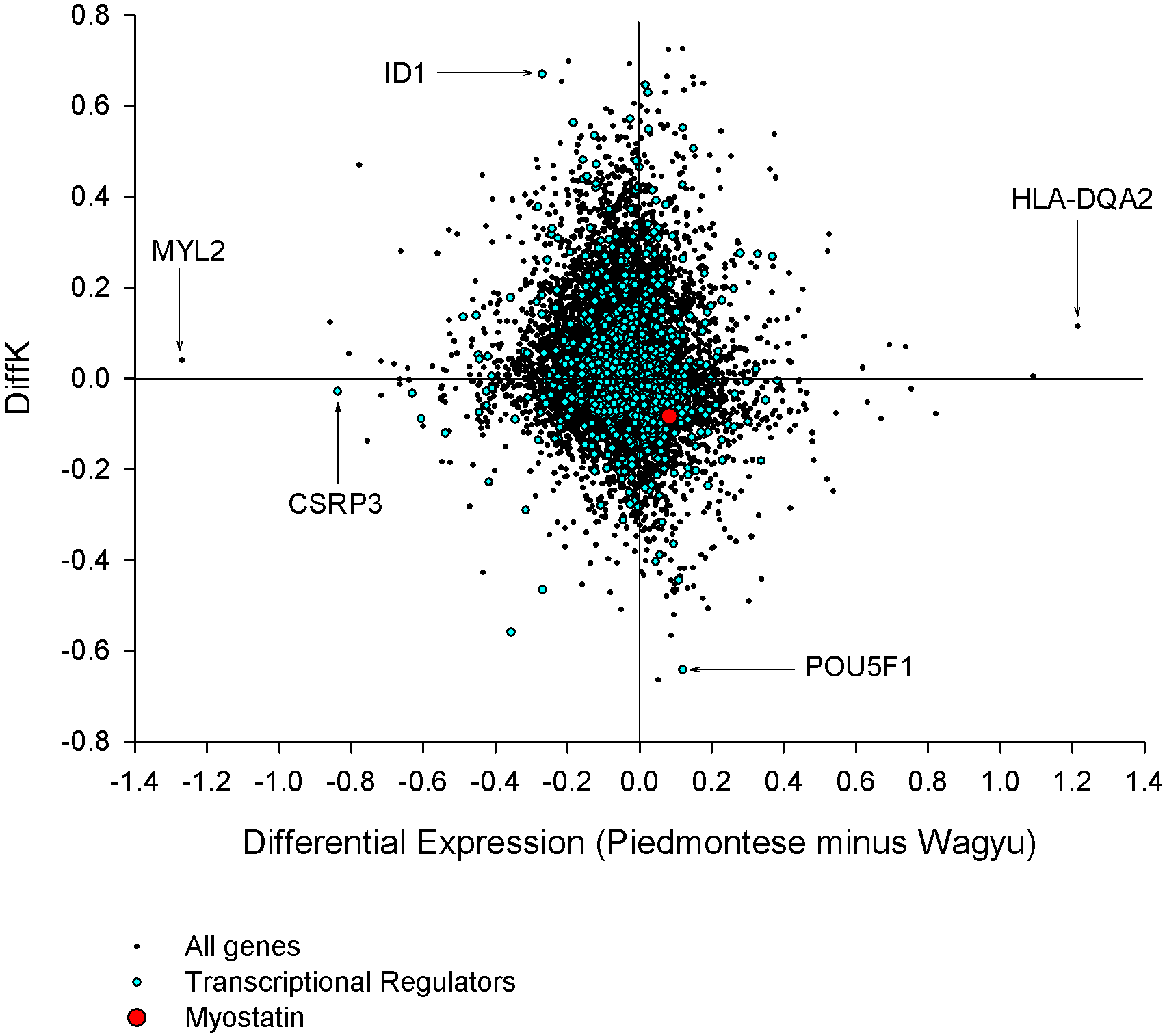

Supplement: Figure S1 — The DE and DiffK for all 11,057 genes. Myostatin is not DiffK. (0.28 MB TIF) [file pcbi.1000382.s001.tif]
